# Supplementary material for: Magnetic excitations in strained infinite-layer nickelate PrNiO2 films
Source: Nat Commun. 2024 Jul 3;15:5576. doi: 10.1038/s41467-024-49940-4 (PMC11220032; doi:10.1038/s41467-024-49940-4)
Supplement: Supplementary file 1 — Supplementary Information [file 41467_2024_49940_MOESM1_ESM.pdf]

Supplementary Information

Magnetic Excitations in Strained Infinite-layer Nickelate $PrNiO_2$ Films

Qiang Gao[1,#], Shiyu Fan[2,#], Qisi Wang[3,4#], Jiarui Li[5], Xiaolin Ren[1,6], Izabela Biało[3,7], Annabella Drewanowski[3], Pascal Rothenbühler[3], Jaewon Choi[8], Ronny Sutarto[9], Yao Wang[10], Tao Xiang[1,6,11], Jiangping Hu[1,6], Ke-Jin Zhou[8], Valentina Bisogni[2], Riccardo Comin[5], J. Chang[3,*], Jonathan Pelliciari[2,*], X. J. Zhou[1,6,12,*], and Zhihai Zhu[1,6,12,*]

[1]Beijing National Laboratory for Condensed Matter Physics,
Institute of Physics, Chinese Academy of Sciences, Beijing 100190, China

[2]National Synchrotron Light Source II,
Brookhaven National Laboratory, Upton, New York 11973, USA

[3]Physik-Institut, Universität Zürich,
Winterthurerstrasse 190, CH-8057 Zürich, Switzerland

[4]Department of Physics, The Chinese University of Hong Kong,
Shatin, Hong Kong, China

[5]Department of Physics, Massachusetts Institute of Technology,
Cambridge, Massachusetts 02139, USA

[6]University of Chinese Academy of Sciences,
Beijing 100049, China

[7]AGH University of Science and Technology,
Faculty of Physics and Applied Computer Science, 30-059 Kraków, Poland

[8]Diamond Light Source, Harwell Campus,
Didcot OX11 0DE, United Kingdom

[9]Canadian Light Source, Saskatoon, Saskatchewan S7N 2V3, Canada

[10]Department of Physics and Astronomy,
Clemson University, Clemson, SC 29631, USA

[11]Beijing Academy of Quantum Information Sciences, Beijing 100193, China

[12]Songshan Lake Materials Laboratory, Dongguan 523808, China

[#]These authors contributed equally to the present work.

(Dated: June 11, 2024)

[*]To whom correspondence should be addressed.

Emails: johan.chang@physik.uzh.ch, pelliciari@bnl.gov, XJZhou@iphy.ac.cn, zzh@iphy.ac.cn

Supplementary Note 1. Sample characterizations

Supplementary Fig. S1a and S1b plot the out-of-plane XRD patterns of ~7 nm thick precursor $PrNiO_3$ films grown on $SrTiO_3$ and LSAT substrates, respectively. Both films show clear out-of-plane (001) and (002) peaks as well as thickness fringes. The (002) peaks locate at 48.5° and 48.0° for the $PrNiO_3$ films grown on $SrTiO_3$ and LSAT, respectively. The in-plane compressive strain applied by the LSAT is expected to be larger than that imparted by $SrTiO_3$ because LSAT has a smaller lattice constant (3.868Å) than that of $SrTiO_3$ (3.905Å). After soft-chemical reduction, the $PrNiO_3$ films were reduced to the infinite-layer $PrNiO_2$, as shown in Supplementary Fig. S1c and S1d. The (002) peaks move to 55.4° and 55.0° for the $PrNiO_2$ film on $SrTiO_3$ and LSAT, respectively. As evidenced in the reciprocal space maps (RSM) of the (103) reflection from two typical $PrNiO_2$ films grown on $SrTiO_3$ and LSAT substrates, shown in Supplementary Fig. S1e and S1f, the films are epitaxially strained on the substrates, similar to the findings for the superconducting $Pr_{0.8}Sr_{0.2}NiO_2$ films grown on $SrTiO_3$ and LSAT substrates [1]. The in-plane lattice constants of the nickelate films highly match those of the substrates, yielding -1% compressive in-plane strain in the films grown on LSAT compared to $SrTiO_3$. It is noticeable that some broad peak weight exists in the reciprocal space maps (RSM) of the (103) reflection in Supplementary Fig. S1f, indicating the film is partly relaxed.

Supplementary Note 2. The momentum resolved *dd* excitations in the $PrNiO_2$ films

In Supplementary Fig. S2, we plot the momentum-resolved *dd* excitations in the $PrNiO_2$ films on $SrTiO_3$ and LSAT substrates. As discussed in the main text, both spectra exhibit four major features corresponding to the crystal field splitting in the energy loss range of 1 - 4 eV and the hybridization between Ni and Pr orbitals at the energy loss of ~0.6 eV. The *dd* excitations of both films are non-dispersive along (*h*, *h*) and (*h*, 0) directions. The momentum dependence of the peak intensity can largely be

accounted for by the matrix elements of the RIXS cross-section, which can be calculated using the single-ion model to determine the orbital character of the *dd* excitations [2]. Previous studies on $NdNiO_2/SrTiO_3$ have shown an excellent agreement between the experimental measurements of the *dd* excitations and the theoretical calculations based on the single-ion model with considering the D_{4h} crystal-field splitting, thus allowing for the assignment of the main peaks to transitions to the specific orbitals [3]. The *dd* intensity map vs momentum transfer and splitting energy for the $PrNiO_2$ films are shown in Supplementary Fig. S2, and they are akin to those for $NdNiO_2$ [3]. We, therefore, assign in the main text the main feature of *dd* excitations in $PrNiO_2$ to the transitions to the orbitals in a similar fashion as in $NdNiO_2$. This assignment also agrees with the studies on $CaCuO_2$, which is isostructural to infinite-layer nickelates [2, 4, 5].

Supplementary Note 3. Fitting of RIXS spectra

Supplementary Fig. S3 shows a typical RIXS spectrum of the $PrNiO_2$ film grown on STO along (h, 0) direction in reciprocal space. The spectrum is fitted with a combination of a Voigt function for the elastic peak, a Gaussian function for the phonon, a damped harmonic oscillator (DHO) to account for the magnetic excitation, and a quadratic function for the weak background. Near the zone boundaries, the magnon and the phonon peaks are well separated, and they can be well-resolved in the fitting, and the phonon is found to be non-dispersive. Near the zone center, the magnon and the phonon peaks overlap substantially with each other. For the spectra taken in this region, we fix the phonon energy and FWHM during the fitting procedure. To estimate the error bars of the magnon energy and damping factor, we vary the phonon intensity during the fitting. We find the phonon intensities vary less than ± 50% to get high-quality fitting results. We also test different functions for the background. We find the linear function and an antisymmetric Lorentz function for the background can also capture the lineshape of the RIXS spectra from -100 to 350 meV. By comparing the best-fit results using different background functions, we were able to estimate the background-induced error bars for the magnon energy and damping factor. The total

error bars are estimated by taking into account the errors from the fitting, different fitting functions, and the phonon intensity variations. In our fitting procedure, we don't perform convolution while fitting since the magnon damping factor is much larger than the energy resolution along with the fact that the phonon energy width is not resolution-limited.

Supplementary Note 4. Low-energy loss RIXS spectra for $PrNiO_2$/LSAT

In Supplementary Fig. S4, we show an enlarged view of the low-energy loss RIXS spectra of Fig. 2c and 2d in the main text. This allows better visualization of the agreement of the fitting to the raw data. Overall, the fit shows a good agreement with the data, enabling us to resolve individual components properly. In Supplementary Fig. S5, we show the low-energy loss RIXS spectra that are collected using π (linear horizontal) polarized incident photons. We have performed a similar fitting procedure to that employed in Supplementary Fig. S4 to resolve individual components in the RIXS spectra. The magnon energies and damping factors for each momentum position obtained from the fitting are shown in Fig. 4b in the main text.

Supplementary Note 5. Linear spin-wave analysis for the spin 1/2 Heisenberg model on the two-dimensional square lattice

The Hamiltonian for the spin 1/2 Heisenberg antiferromagnet on the square lattice is given by

$$H = J_1 \sum_{\langle ij \rangle} S_i \cdot S_j + J_2 \sum_{\langle ii' \rangle} S_i \cdot S_{i'} \tag{1}$$

where $J_1(J_2)$ represents the exchange coupling for nearest-neighbours (next-nearest neighbours), S_i is the spin-1/2 operator on site i, and $\langle ij \rangle$ ($\langle ii' \rangle$) denotes the nearest-neighbours (next-nearest neighbours) [6]. Using the linear spin wave theory, the magnetic dispersion is

$$\omega = 2Z_c \sqrt{A_q^2 - B_q^2} \tag{2}$$

Where

$$A_q = J_1 - J_2[1 - \cos(2\pi h)\cos(2\pi k)] \quad (3)$$

$$B_q = J_1[\cos(2\pi h) + \cos(2\pi k)]/2 \quad (4)$$

In our simulation, the renormalization factor Zc was fixed at 1.18, which is expected for a 2D spin-1/2 square-lattice Heisenberg AFM [6].

Supplementary Note 6. Test of tolerance in fitting by fixing the magnon peak position to scale with an increase of 40% in Tc

As shown in Fig. 4a in the main text, the magnon bandwidth for $PrNiO_2$/LSAT is slightly smaller than that for $PrNiO_2$/STO. This is further confirmed in the measurement using different scattering geometry with π polarized incident photons, as summarized in Fig. 4b in the main text. In the case of the σ and grazing incidence geometry, the data shown in Fig. 2c and 2d in the main text (enlarged in Supplementary Fig. S4) appear not as good as those for $PrNiO_2$/STO. In the fitting procedure, multiple components were used in the fitting to achieve a good agreement to data, as shown in Supplementary Fig. S4. Nonetheless, it is still necessary to test the tolerance in the fitting by fixing the magnon peak position to scale with an increase of 40% in Tc. Near the Brillouin zone boundary (*h, k*) = (0.5, 0) and (0.25, 0.25), the magnon peak position is approximately proportional to the magnon bandwidth. Supplementary Fig. S6a and S6b show, respectively, the low energy loss RIXS spectra at (0.47, 0) and (0.25, 0.25) obtained using σ polarized incident photons. The fit is given by the solid red curve, which shows good agreement with the data. In Supplementary Fig. S6c and S6d, we fix the magnon peak positions with an enhancement of 40%. This yields a substantial deviation between the fit (solid red curves) and the data. We have performed a similar analysis for the data measured using π polarized incident photons. As shown in Supplementary Fig. S7c and S7d, the solid red curves represent the fit to the data with the magnon peak positions fixed to scale

with a 40% increase to the corresponding values shown in Supplementary Fig. S7a and S7b; likewise, they deviate substantially from the measurements.

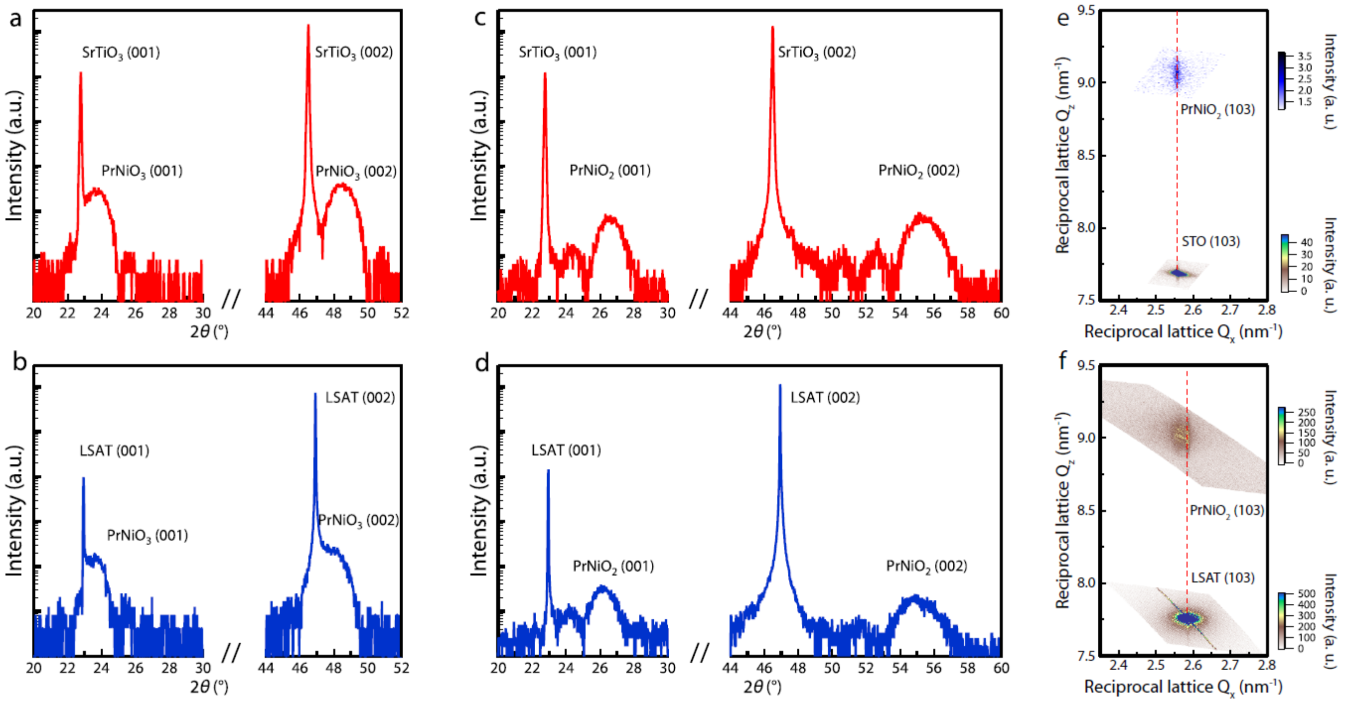

Supplementary Fig. S1: X-ray diffraction pattern of the nickelate films grown on different substrates. a, b, The precursor phase $PrNiO_3$ films grown on $SrTiO_3$ and LSAT substrate. c, d, The infinite-layer $PrNiO_2$ films on $SrTiO_3$ and LSAT substrate. e, f, Reciprocal space maps (RSM) collected around the (103) reflections from two typical $PrNiO_2$ films on $SrTiO_3$ and LSAT substrates.

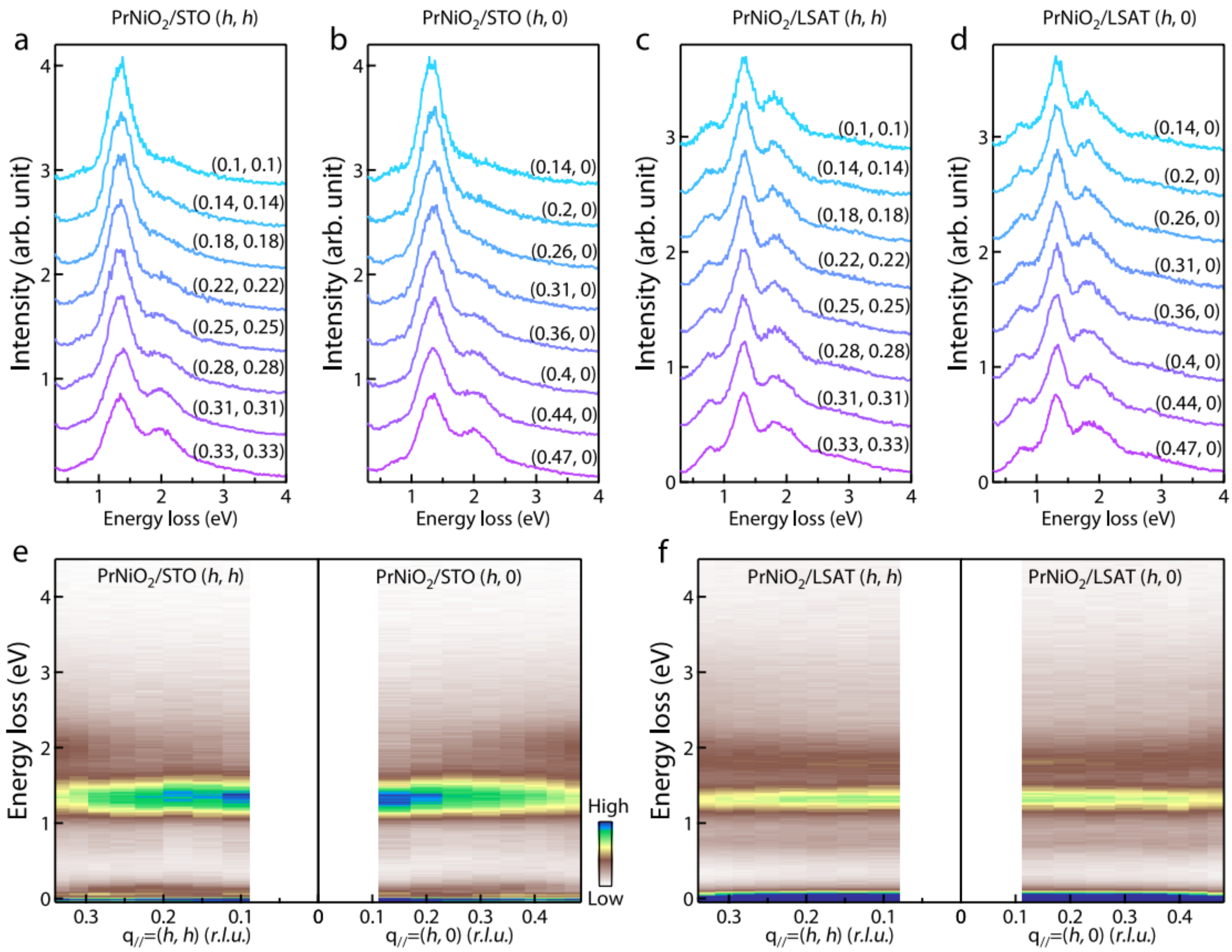

Supplementary Fig. S2: The momentum resolved *dd* excitations of the $PrNiO_2$ films along high symmetric directions. a,b, The *dd* excitations of the $PrNiO_2$ film grown on $SrTiO_3$ substrate along (*h*, *h*) and (*h*, 0) directions. c,d, The *dd* excitations of the $PrNiO_2$ film grown on LSAT substrate along (*h*, *h*) and (*h*, 0) directions. e,f, RIXS maps of the *dd* excitations of the $PrNiO_2$ films grown on $SrTiO_3$ and LSAT substrate.

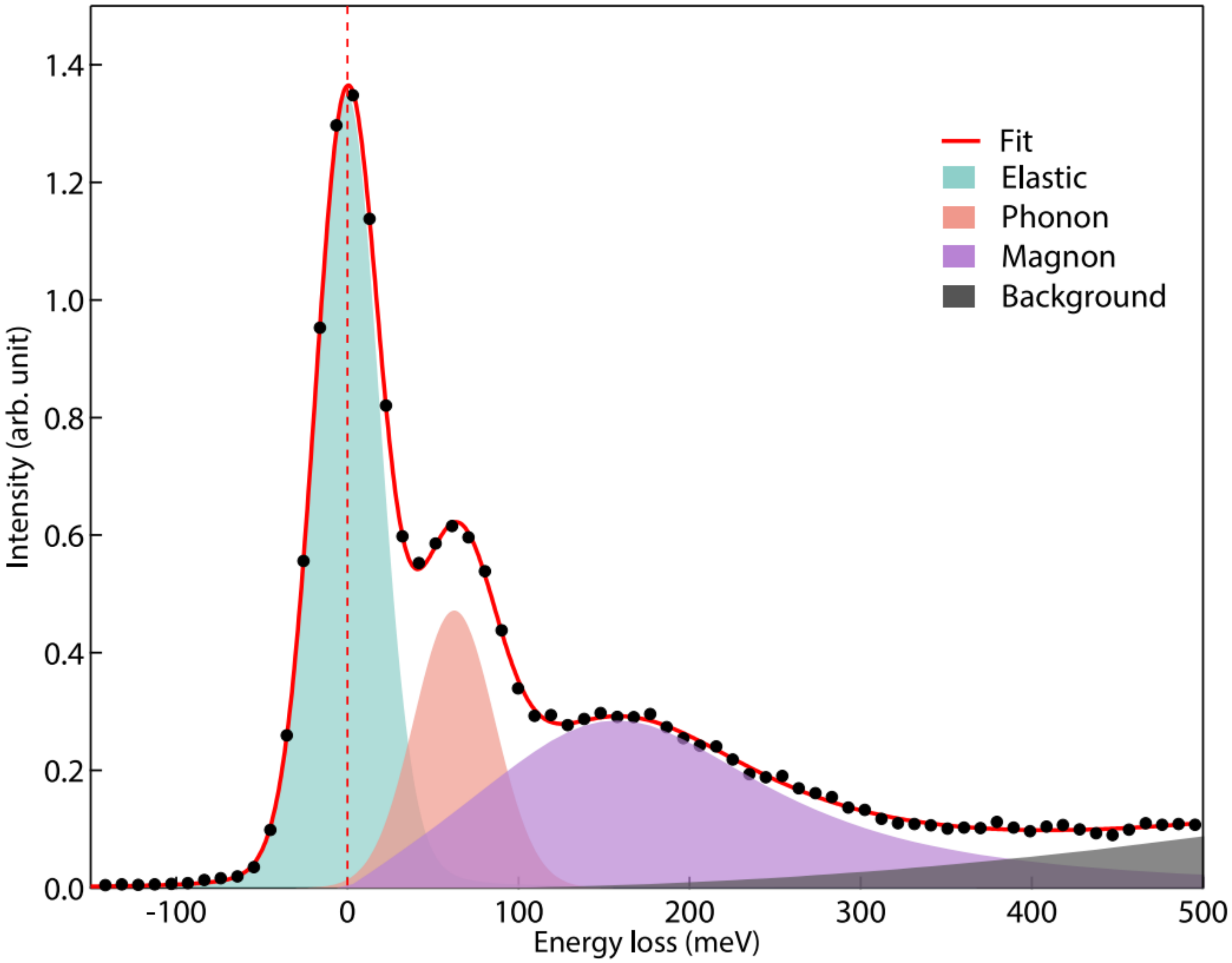

Supplementary Fig. S3: RIXS spectra of the $PrNiO_2$ film grown on STO along (*h*, 0) direction at zone boundary with a momentum transfer of (0.47, 0). The filled black circles represent the data and the solid red curves fit the data set, using a combination of an elastic scattering contribution (green), a Gaussian profile for the phonon peak (orange), a DHO function for the magnetic excitation (purple), and background (grey).

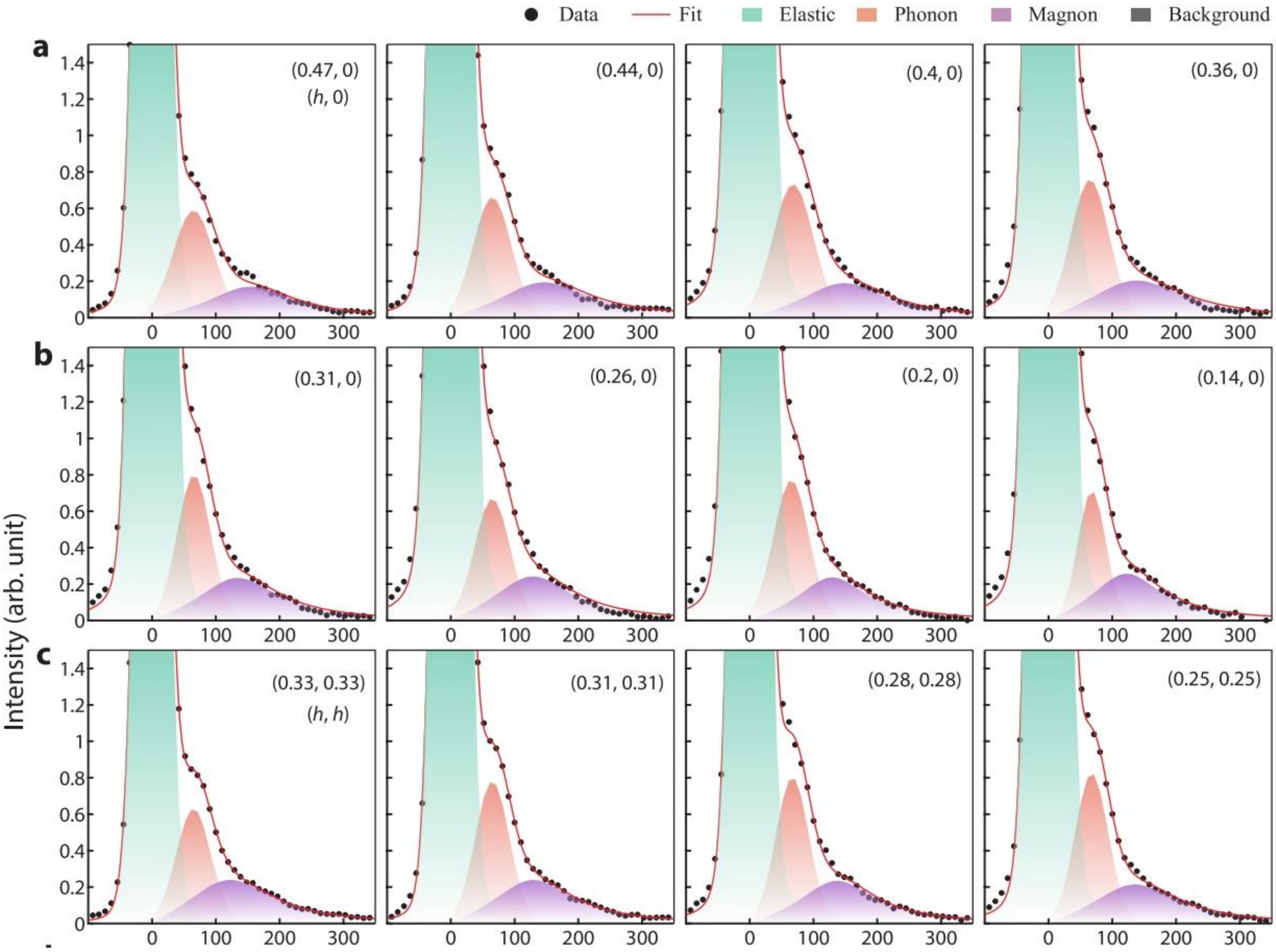

Supplementary Fig. S4: Momentum resolved RIXS spectra along high symmetry directions. RIXS spectra of the $PrNiO_2$ film grown on LSAT along (*h*, 0) and (*h*, *h*) directions. The filled black circles represent the data and the solid red curves fit the data set, using a combination of an elastic scattering contribution (green), a Gaussian profile for the phonon peak (orange), a DHO function for the magnetic excitation (purple), and background (grey). All the measurements were taken at 40 K with σ polarized incident photons.

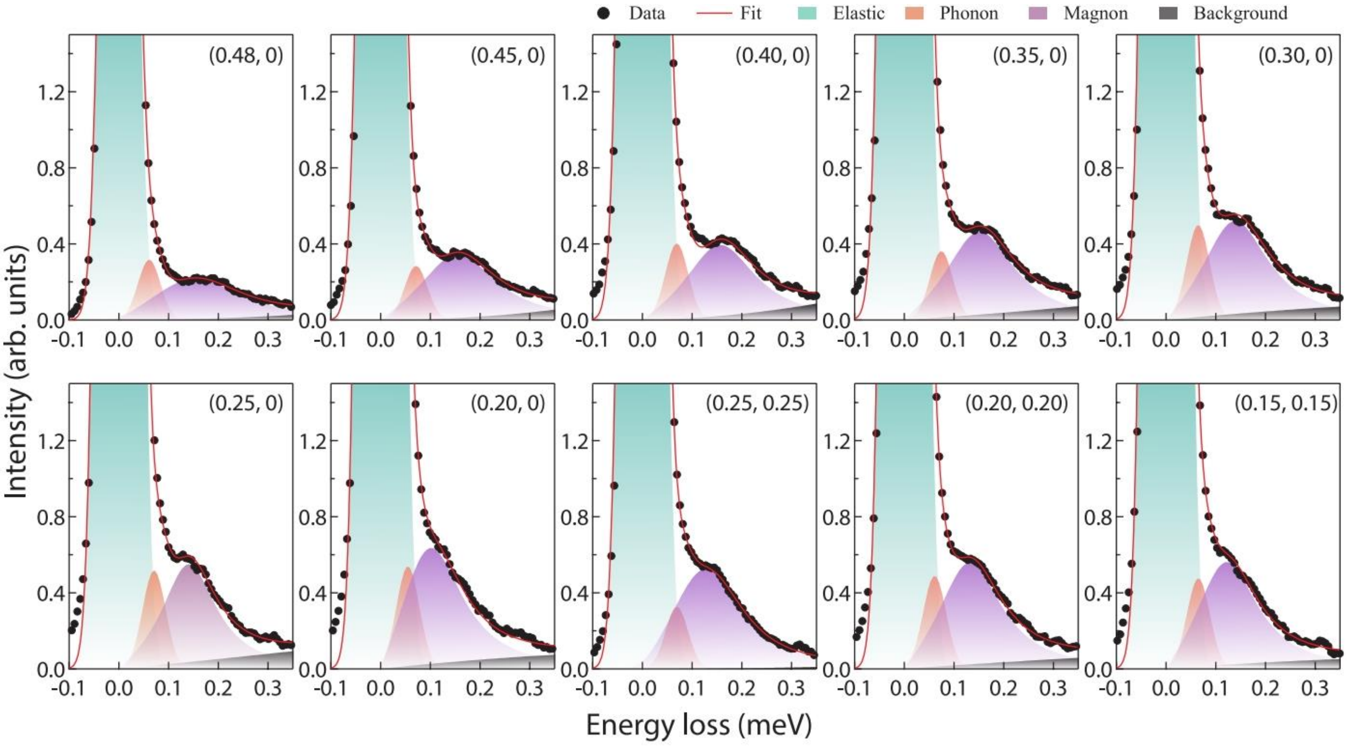

Supplementary Fig. S5: Momentum resolved RIXS spectra along high symmetry directions. RIXS spectra of the $PrNiO_2$ film grown on LSAT along (h, 0) and (h, h) directions. The filled black circles represent the data and the solid red curves fit the data set, using a combination of an elastic scattering contribution (green), a Gaussian profile for the phonon peak (orange), a DHO function for the magnetic excitation (purple), and background (grey). All the measurements were taken at 16 K with π polarized incident photons.

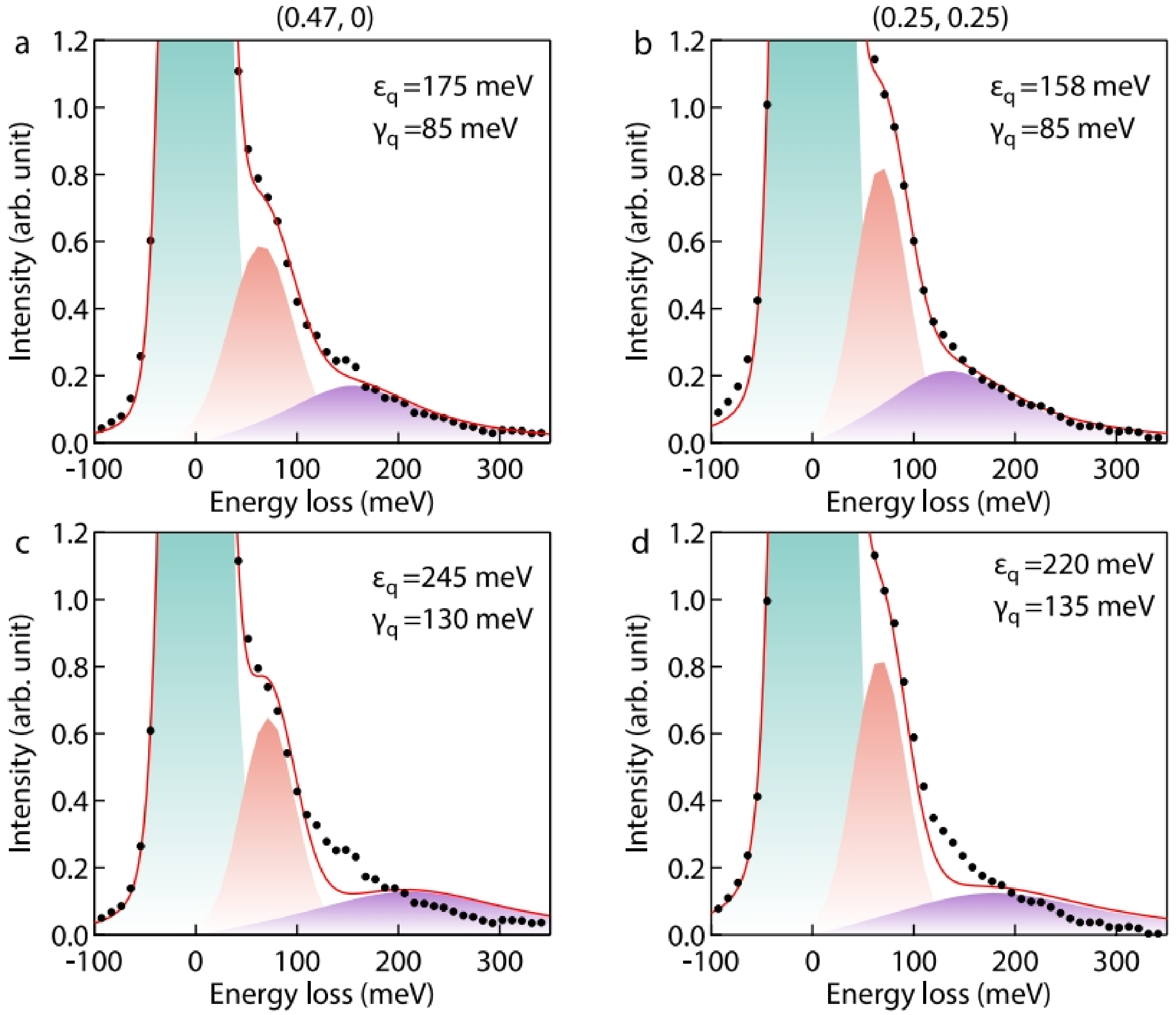

Supplementary Fig. S6: Fitting the RIXS spectra with the magnon position fixed to scale with an increase of 40% in Tc. a and b show respectively the RIXS spectra at (0.47, 0) and (0.25, 0.25) measured using σ polarized incident photons. The filled black circles represent the data and the solid red curves fit the data set. c and d represent the same data set corresponding to a and b, but the solid curves are the fits with magnon peak position fixed.

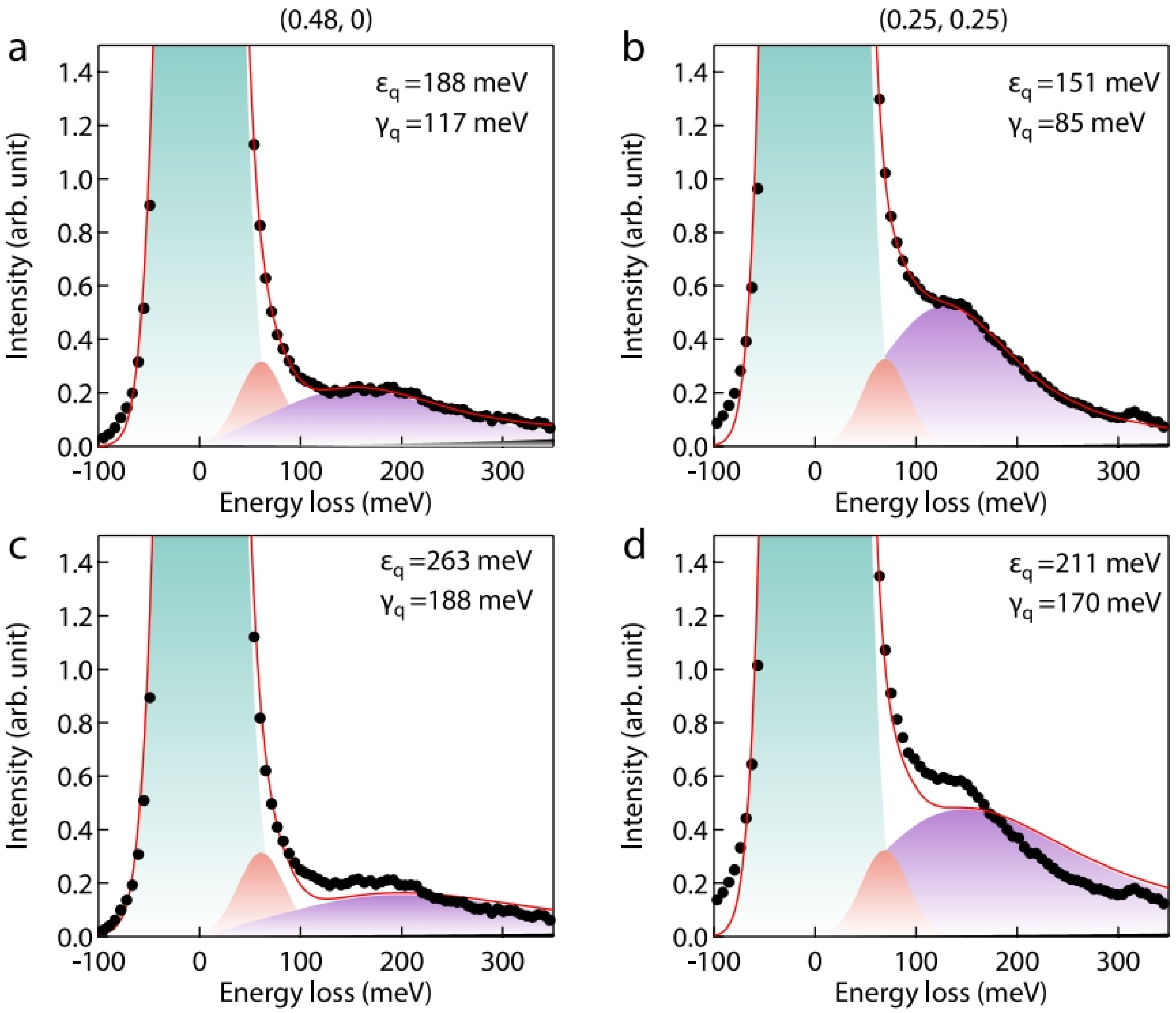

Supplementary Fig. S7: Fitting the RIXS spectra with the magnon position fixed to scale with an increase of 40% in Tc. a and b show respectively the RIXS spectra at (0.48, 0) and (0.25, 0.25) measured using π polarized incident photons. The filled black circles represent the data and the solid red curves fit the data set. c and d represent the same data set corresponding to a and b, but the solid curves are the fits with magnon peak position fixed.

Supplementary References

1 Ren, X. L. et al. Strain-induced enhancement of T_c in infinite-layer $Pr_{0.8}Sr_{0.2}NiO_2$ films. *Commun Phys* **6**, 341 (2023).

2 Sala, M. M. et al. Energy and symmetry of *dd* excitations in undoped layered cuprates measured by Cu L_3 resonant inelastic x-ray scattering. *New J. Phys.* **13**, 043026 (2011).

3 Anderson, Rossi, M. et al. Orbital and spin character of doped carriers in infinite-layer nickelates. *Phys. Rev. B* **104**, L220505 (2021).

4 Minola, M. et al. Measurement of the effect of lattice strain on magnetic interactions and orbital splitting in $CaCuO_2$ using resonant inelastic x-ray scattering. *Phys. Rev. B* **87**, 085124 (2013).

5 Hozoi, L. et al. Ab Initio determination of Cu 3*d* orbital energies in layered copper oxides. *Scientific Reports* **1**, 65 (2011).

6 Coldea, R. et al. Spin Waves and Electronic Interactions in La_2CuO_4. *Phys. Rev. Lett.* **86**, 5377 (2001).
